# Long-Term Climate Sensitivity of Grazer Performance: A Cross-Site Study

**DOI:** 10.1371/journal.pone.0067065

**Published:** 2013-06-20

**Authors:** Joseph M. Craine

**Affiliations:** Division of Biology, Kansas State University, Manhattan, Kansas, United States of America; University of Alberta, Canada

## Abstract

Climate change will affect grasslands in a number of ways, but the consequences of a warmer, drier world for grazers is uncertain. Predicting future grazer performance is complex since climate change affects both the quantity and quality of forage through a combination of processes that occur over a range of time scales. To better predict the consequences of climate change for grazer performance, a dataset was compiled of over a quarter million bison weights distributed across 22 US herds that span a large range of climates. Patterns of bison body mass among sites, age classes, and sexes were analyzed with respect to differences in geographic patterns of climate and interannual variation in climate. While short-term effects of climate variability are likely to depend on the magnitude and timing of precipitation during the year, grazers will be negatively affected by sustained hotter, drier conditions most likely associated with reductions in forage quality. Short-term, little effect of high temperatures on bison performance is observed, which suggests that the long-term effects of higher temperatures are likely to accrue over time as nitrogen availability in grasslands is reduced and forage quality declines. If relationships observed for bison are general for cattle, the economic consequences of higher temperatures due to decreased weight gain in US cattle could be on the order of US$1B per 1°C increase in temperature. Long-term monitoring of forage quality as well as native and domesticated grazer performance is recommended to better understand climate change effects on grazers.

## Introduction

Over the coming century, mean annual temperatures are predicted to increase globally by 2–7°C while regions such as the North American Great Plains may experience increased or decreased precipitation [1], [2]. Climate change is likely to affect the growth and reproduction of domestic grazers like cattle as well as native grazers such as bison in North America due in part to the effects of climate on forage quantity and quality [3], [4]. Because of their pivotal role in grassland function [5]–[8], changes in the performance and ecology of grazers would likely have substantial effects on the functioning of grasslands, but also have the potential to incur substantial economic costs. Despite the potential importance of climate change on grazer performance, predictions of how climate change would affect grazer performance have been ambiguous [9].

Although multiple experiments have investigated the role of climate change on grasslands, almost all grassland climate change experiments exclude large grazers, which limits the ability of the experiments to predict how climate change will affect grazers. Grazers increase water, light, and nutrient availability [10], [11] and strongly affect grass productivity, species composition, and plant quality [5], [12]. As a result, the presence of grazers can generate alternative stable states that could reverse the effects of climate change on ecological components [13], [14]. The fundamental differences in grazed and ungrazed grasslands weaken predictions regarding the consequences of climate change for grazers from experiments without them.

With the restricted utility of grassland climate change experiments, predicting climate change effects on grazers requires assessing how grazers respond to interannual and geographic patterns of climate [15], [16]. While each approach has its limitations, quantifying grazer responses to interannual climate variation indexes short-term responses of the grazer-grassland system to climate variability, while geographic patterns index long-term responses that incorporate slower processes such as shifts in plant community composition and soil organic matter dynamics in ways that are useful analogs for future climates [17], [18].

Interannual variation in climate can affect grazers in multiple ways [19]–[21], but the degree to which short-term variability in climate will preface responses to long-term shifts are unclear. Previous investigations of geographic patterns of herbivore biomass generate predictions that the total biomass of large mammalian herbivores would decrease with decreasing precipitation [22], [23], although the consequences of changes in precipitation are likely to depend on soil fertility [24], [25]. Increasing temperatures have the potential to select for larger or smaller animals [26], but the consequences of warming for growth rates is even less well known than the eventual net effect.

Due to the role of grazers such as cattle and bison on the ecology on grasslands as well as their economic importance, there is a need to better understand how climate change is likely to affect the performance of grazers. In order to investigate how climate change will affect grazers in North America, a dataset was compiled that included over 290,000 body mass of bison (*Bison bison*) distributed across 22 US herds (Table 1). Herds were distributed across a bioclimatic range of more than 11°C mean annual temperature (MAT) and 600 mm of mean annual precipitation (MAP). The restricted genetic differentiation of the bison herds [27] minimizes confoundedness between bison genetics and the climate gradients. Accounting for the sex and age of each individual, relationships between geographic patterns in mean climate and bison performance were examined, while the responses of bison performance to interannual variation in climate were compared for three sites using the critical climate period approach [28], [29].

**Table 1:** Summary information for bison herds.

Level	Lat.	Long.	Elev (m)	MAP (mm)	Precip_June_ (mm)	MAT (°C)	Years
Antelope Island	41.06	−112.24	1322	333.8	29.2	10.70	1993–2010
Badlands	43.81	−102.51	853	344.9	81.3	8.43	1998–2010
Bad River	44.21	−100.74	499	337.2	77.0	8.42	2004–2011
Blue Creek	41.63	−102.16	1158	381.7	79.2	8.99	2004–2010
Custer	43.72	−103.40	1327	357.3	76.0	7.46	2005–2010
Deer Creek	42.56	−102.24	1158	375.0	81.4	8.39	2004–2010
Fawn Lake	42.45	−101.83	1128	373.3	81.8	8.44	2007–2010
Flying D	45.61	−111.44	1767	354.4	68.5	6.62	2001–2011
Konza	39.10	−96.61	335	704.9	132.5	12.58	1994–2011
McGinley	43.00	−101.94	1052	356.2	81.7	8.31	2004–2010
Nat. Bison Range	47.32	−114.21	1310	363.8	59.8	6.81	1998–2011
Ft. Niobrara	42.88	−100.45	744	400.8	78.6	8.36	1987–2011
Ordway	45.71	−99.10	579	395.4	84.0	5.70	2004–2011
San Luis Valley	37.80	−105.71	2316	201.6	21.4	6.30	2008–2011
Snowcrest	45.05	−112.11	1829	353.8	61.6	4.38	2004–2010
Spikebox	42.41	−101.22	1012	393.2	81.9	8.48	2004–2011
Tallgrass Prairie	36.75	−96.34	274	797.3	118.2	14.45	1995–2011
T. Roosevelt	47.57	−103.29	720	312.4	80.1	5.42	1985–2008
Vermejo	36.83	−104.85	2255	380.5	43.2	6.00	2002–2010
Wichita Mtn	34.77	−98.67	639	589.1	87.5	15.71	2008–2011
Wind Cave	43.58	−103.47	1280	349.4	73.2	7.49	1983–2009
Z Bar	37.11	−98.93	529	545.0	102.2	13.72	1999–2011

Data include latitude and longitude (decimal degrees), elevation, mean annual precipitation (MAP), mean June Precipitation, mean annual temperature (MAT), and the year range for the mass measurements.

## Methods

Data on bison mass were acquired from original sources. Only masses where the sex of each animal had been identified and its age could be calculated were included in analyses here. Bison ages were generally determined directly from tagging of individual calves and yearlings and recensusing them over time. Only individuals that were weighed between September 15 and January 30 were included and average masses were calculated for any individual weighed twice during this period in a year. Date weighed explained less than 0.5% of the variation in individual masses. 411 individuals were removed from the final dataset. These were animals with masses that were more than three standard deviations from the mean for a given sex-age class and/or were calves less than 75 kg, which indicates either errors in weighing or late-born individuals. As the number of animals present in herds declines with age due to natural mortality and management practices-for example some sites do not allow males older than 7 years of age to remain in the herd-masses from females older than 12.5 y and males older than 6.5 y were also excluded. The final data set included 296,171 masses, of which 67% were female. Each herd was weighed an average of 10.5 times. Animals were not supplemented nutritionally outside of minerals. Ages of the youngest animals were assumed to be 0.5 y with intervals of 1 y for older animals since birth dates were not recorded for most animals. The average individual was weighed 2.4 times in the dataset.

To ultimately determine the relationships between climate and bison mass, a two-stage analysis was used. First, mean body mass of each sex standardized for age was determined for each herd. Second, relationships between climate and standardized body mass were tested. To derive a standardized body mass, mean body mass was calculated for each combination of nominal age, sex, and site. A linear regression model was then run that predicted body mass with age (categorical) and site (random effects) for each sex:
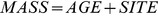
(eq.1)


Least squares means were generated for each sex at each site, which generated age-standardized masses for males (3.5 y) and females (6.5 y) among sites.

To test for relationships between standardized body mass and climate, mean annual and mean monthly temperatures and precipitations were acquired for each site from New et al. [30]. Forward-elimination stepwise regression (*P*<0.01) was used to select the climate variables that significantly predicted variation in standardized masses of bison among sites for each sex. A subsequent model tested the role of the two significant climate parameters (mean annual temperature [MAT] and June precipitation [Precip_June_]) and sex on standardized body mass:
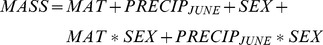
(eq.2)


To examine how the significant climate predictors of body mass (MAT and June Precipitation) affected bison of different ages, individual regressions were run using MAT and June precipitation to predict variation in body mass for each age for males and females.

The effects of short-term variation in climate had been assessed for two sites earlier (Konza and Tallgrass Prairie Preserve [29] and only one additional site (National Bison Range) had data for enough years and access to daily climate data to assess this. In order to assess the effects of short-term climate variation on body mass, critical climate period analysis [28], [29] was performed for National Bison Range bison using forward selection stepwise regression on all combinations of average temperature and summed precipitation from March 1 – October 2 with a minimum of 15-d windows and 5-d increments. Analyses were conducted for age-standardized masses of sexes from previous analyses as well as calf masses. Maximum P-value for inclusion in the final model was 0.01. Climate data for the National Bison Range critical climate period analysis was acquired from nearby St. Ignatius, MT for this time period and downloaded from www.knmi.nl.

To compare the relative effects of climate on bison mass as forage quality [4], an additional model of bison mass was run that included MAT and MAP, which were the two climate variables that explained a high proportion of variation in geographic patterns of the forage quality of cattle [4].

Calculations of potential economic costs of 1°C warming to the US cattle industry were generated from the product of the MAT effect on masses (−10.9 kg/°C), the market price for live cattle (US$2.64/kg) (www.ams.usda.gov/LSMarketNews, accessed May 14, 2012), and the number of cattle slaughtered in the US in 2011 (34.1M) [31]. Standardized weight of bison associated with the −10.9 kg/°C relationship was 473 kg, which is 19% less than the average live weight of cattle brought to slaughter in the US (582 kg, www.nass.usda.gov).

All statistical analyses were performed in JMP 9.0.3.

## Results

**Figure 1:**
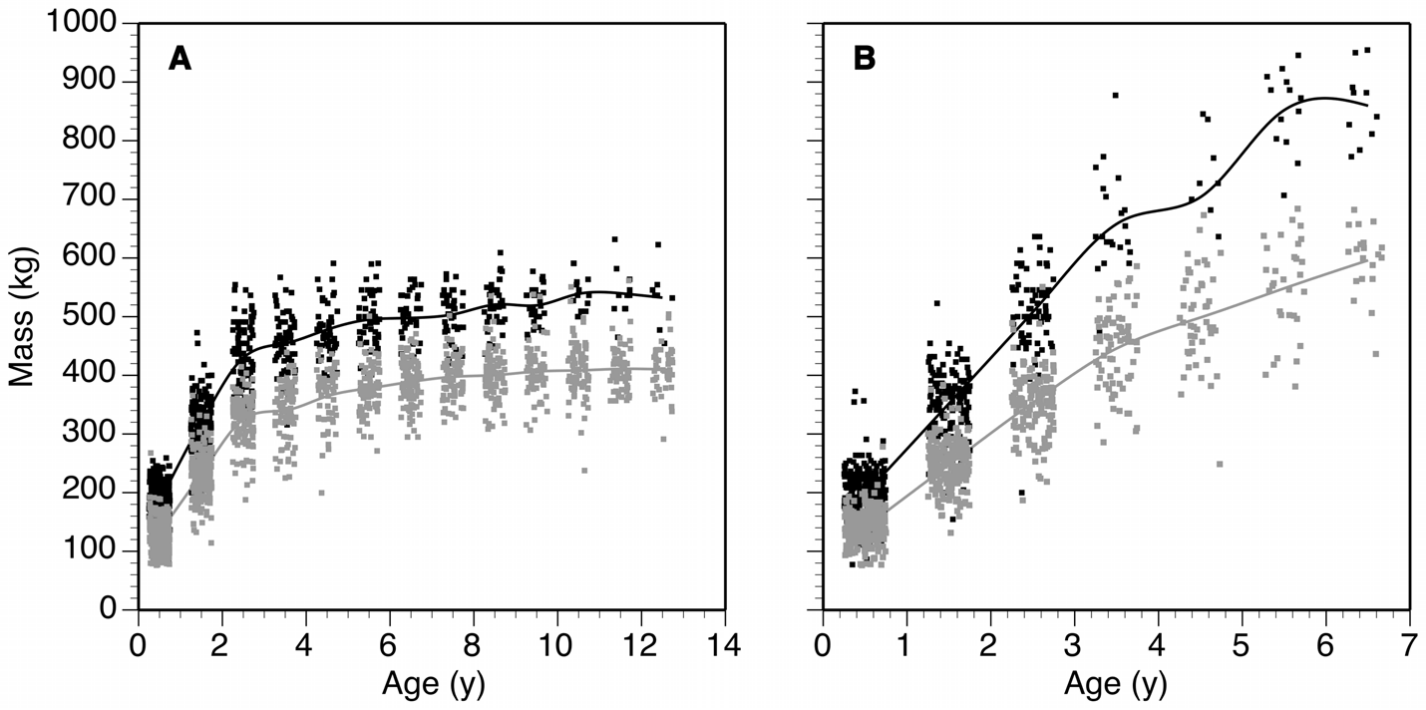
Growth curves for bison. Shown are female (a) and male (b) bison from Wichita Mountains, Oklahoma (grey) and Ordway Prairie, South Dakota (black). Unconstrained spline fit to mean mass of each age cohort for each site shown. Ages are jittered to show point density.

Across the 22 sites, male bison calves averaged 164.5±0.3 kg and female bison calves averaged 156.9±0.3 kg. By 6.5 y, females weighed 448.7±0.4 kg and males 736.4±1.4 kg. The oldest cohort of female bison that was examined (12.5 y) averaged 462.9±0.7 kg. Sites with heavier male bison also had heavier female bison (y = 60.3+0.65x; CI = 0.51–0.83; Pearson's *r* = 0.93, *P*<0.001). For example, 6.5-y old Wichita Mountains, Oklahoma female bison weighed 389 kg while Ordway Prairie, South Dakota female bison of the same age weighed 498 kg (Fig. 1a). 3.5-y old Wichita Mountains male bison averaged 446 kg as opposed to 658 kg at Ordway Prairie (Fig. 1b). By 6.5 y, the difference in masses of male bison between the two sites averaged 260 kg (596 vs. 856 kg).

**Figure 2:**
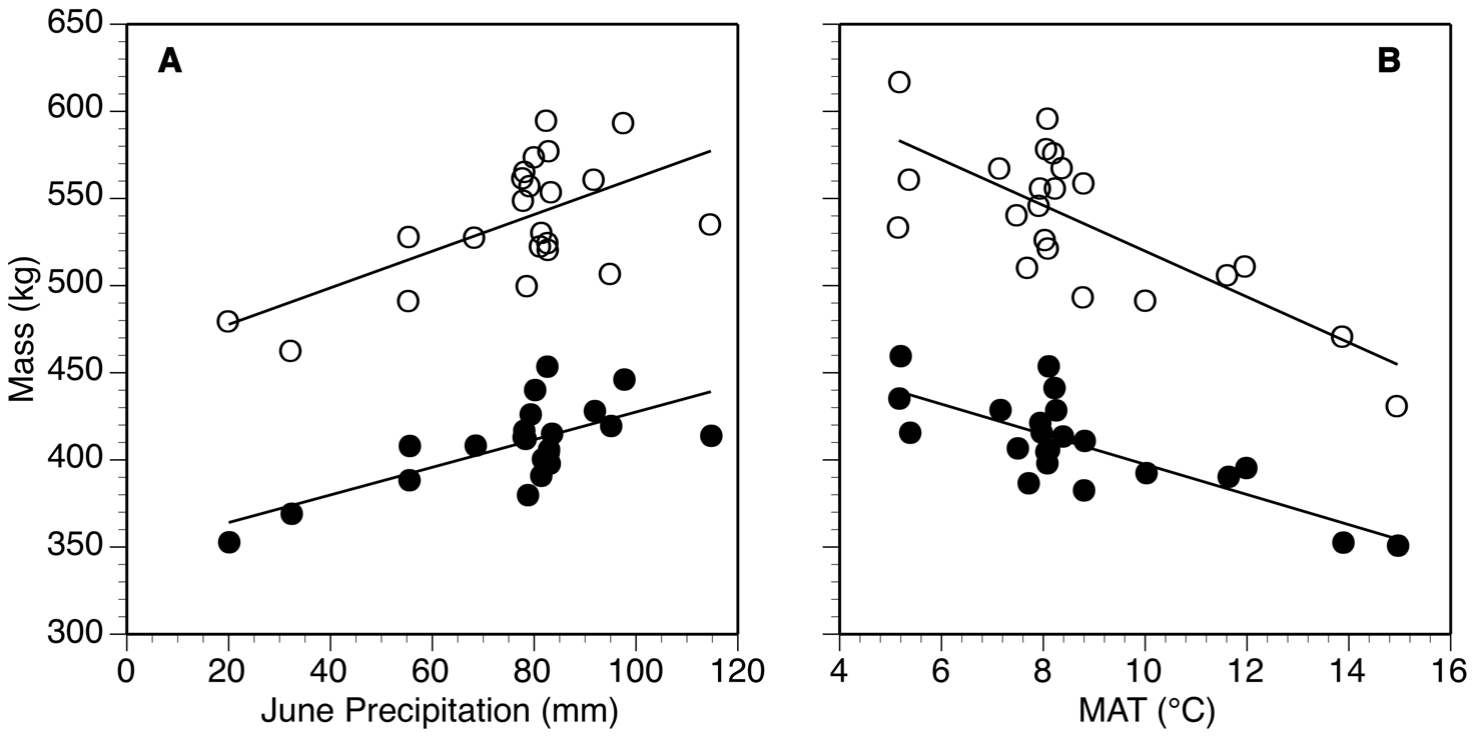
Patterns of body mass across climate gradients. Partial residual plots for standardized body mass and (a) mean June precipitation and (b) MAT across 22 herds. Separate lines for males (open circles) and females (solid circles).

**Figure 3:**
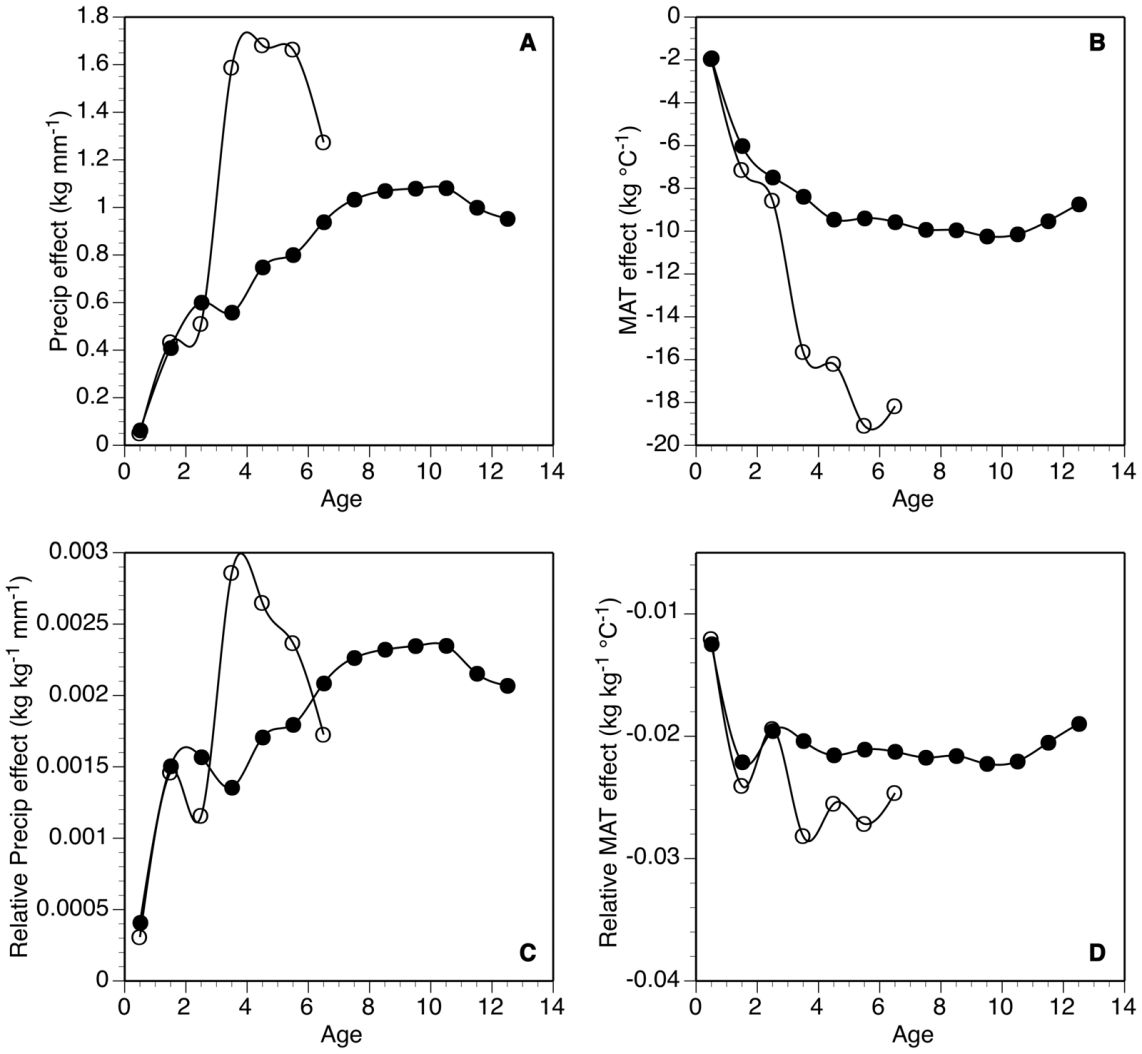
Patterns of bison mass with age. Age-specific sensitivity of bison mass to variation in climate calculated on an absolute basis (a,b) and relative to the average mass of individuals at a given age (c,d) for June precipitation (a,c) and mean annual temperature (b,d). Smoothing splines used to connect individual points.

With variation among herds as much as 100 kg for females and 250 kg for males at a given age, geographic patterns of bison mass suggest that increases in MAT at a site would decrease grazer mass (Fig. 2). For every 1°C increase in MAT, bison mass declined −13.1±2.6 kg °C^−1^ for males and −8.6±1.6 kg °C^−1^ for females (*P*<0.001 for both). Greater MAT had a larger effect on older bison, both on an absolute and a relative basis (Fig. 3). For example, increasing MAT 1°C decreased mass of female calves by 1.9±1.2 kg, but 9.6±1.7 kg for 6.5-y old adult females (Fig. 3).

In addition to increased temperature being associated with lower bison weight, bison in arid areas were lighter than those from wetter regions (Fig. 2). For every 1 mm of mean June precipitation (the best predictor of mass assessed via forward elimination regression) decrease across sites, female bison declined 0.79±0.19 kg and males 1.05±0.3, *P*<0.001 for both). Older bison were more affected by lower mean June precipitation than younger bison, both on an absolute and relative basis (Fig. 3). For example, lowering June precipitation decreased mass of female calves by only 0.06±0.15 kg mm^−1^, but 0.94±0.20 kg mm^−1^ for 6.5 y old adult females.

While geographic patterns of bison performance suggest that increases in temperature and decreases in precipitation will reduce the performance of grazers, analyses of bison performance responses to interannual variation suggest that long-term responses to climate changes are decoupled from short-term responses. Across 17 years at Konza Prairie, Kansas (MAP = 705 mm; MAT = 12.6°C) and 12 years at Tallgrass Prairie Preserve, Oklahoma (MAP = 797 mm; 14.5°C), bison mass gain was greater in years with greater late summer precipitation (August), lower in years with greater mid-summer precipitation (mid-June – mid-July), but unaffected by interannual variation in temperature at different times of year [29]. At the National Bison Range, Montana (MAP = 363 mm; MAT = 6.8°C), from 1998–2010, age-adjusted mass increased with increasing midsummer precipitation for females (June 19 – August 3; *P* = 0.007) at a rate of 0.35±0.11 kg mm^−1^ (Table 2), with a similar trend for males over the same period (0.51±0.22 kg mm^−1^; *P* = 0.04). This general pattern was not restricted to adults as greater mid-summer precipitation also led to heavier female and male calves (critical climate period = June 19 – July 9; 0.55±0.17 kg mm^−1^, *P* = 0.008 for females; 0.64±0.18 kg mm^−1^, *P* = 0.006 for males). Interannual variation in temperature did not explain any significant variation in mass beyond what was explained by precipitation.

**Table 2:** Summary of critical climate period analysis for National Bison Range bison from 1998–2010.

	Parameter	Estimate	P
All-Female	Intercept	402.0±4.9	<0.001
	Precip_170–214_	0.35±0.11	0.007
Calf-Female	Intercept	115.2±5.1	<0.001
	Precip_170–189_	0.55±0.17	0.008
Calf-Male	Intercept	118.9±5.5	<0.001
	Precip_170–189_	0.64±0.18	0.006

There were no significant predictors (*P*<0.01) of male bison weight when using all age classes (0.5–6.5y).

Comparing the effect of a 100-mm decline in mean annual precipitation with a 1°C increase in MAT, MAP and MAT had similar effects on bison mass (−14.0±0.7 vs. −11.6±3.3 kg; ratio = 1.21). Yet, comparing the effects of equivalent changes in MAP and MAT for dietary crude protein on pastures [4], MAP had twice the effect on grass protein as MAT (−6.03 vs. −2.78 mg g^−1^ maximum crude protein; ratio = 2.15).

## Discussion

Long-term shifts in precipitation and temperature likely will affect bison mass through changes in quantity of forage produced, but also the dietary quality of forage. The strong geographic patterns of bison mass parallel the changes in grass nutritional quality that occur across North American grasslands that are grazed by cattle [4], [32]. Drier, hotter regions have lower forage quality, just as bison weight declines as mean climate becomes drier or hotter. Yet decreases in MAP are associated with a proportionally greater reduction in bison mass than they do in forage quality, which could implicate changes in quantity of grass restricting growth, for example, or non-linearity in the effect of reductions in dietary quality on bison mass. The equivalency of 100 mm of precipitation and 1°C increase in mean annual temperature for bison mass suggests that unreasonably large increases in precipitation would be required to balance projected increases in temperature.

There is the potential that the relationships between climate and bison mass are not ultimately caused by climate and instead are influenced by factors that happen to be correlated with climate. For example, the geographic patterns in bison mass might be caused by grazing pressure relative to production being higher in hot, dry ecosystems than in cold, wet ecosystems. Yet, due to the climate relationships with grass productivity and the difficulty in also accounting for the amount of grazable land, actual grass production rates, and the consumption of forage by other wildlife species such as prairie dogs (*Cynomys* sp.) and elk (*Cervus canadensis*), trying to separate these factors statistically is not feasible. Given the strong relationships between climate and forage quality for cattle as well as the observation that the herds in this study seem to be sustainable long-term, having the relationships between bison mass and climate being caused by, for example, geographic patterns of overgrazing seem to be a less parsimonious explanation at this time compared to the role of climate determining forage quantity and quality. That said, more controlled studies of bison grazing in replicated experiments and monitoring of dietary forage quality of bison across geographic gradients would help to further our understanding of the short- and long-term effects of climate variability on bison weight gain.

The differences in short- and long-term effects of temperature on bison mass might represent the relatively slow speed at which temperature affects N cycling and plant N concentrations, which are central to forage quality. For example, warming across four grassland systems initially increased primary productivity, but these effects declined over 9 years as accelerated N losses accumulated and plant species shifted to dampen initial responses [33]. Accelerated N losses and reduction in soil organic matter quality appear to be consistent consequences of long-term warming [34] and have been paired with long-term experimental warming reducing plant N concentrations [35]. As such, long-term warming might drive reduced weight gain if not population numbers [36] by reducing soil N availability and subsequently forage quality. If variation in forage quality is driving geographic patterns in grazer mass, then it is possible that the long-term cumulative effects of higher temperatures on N availability could be driving reduced forage quality and ultimately reducing weight gain.

The effects of future climate change on grazers will be a mix of short-term and long-term responses of grasslands, with the decadal-scale consequences depending on the rates of climate change and rates at which climate change feeds back to factors such as forage quality. Geographic patterns are an imperfect guide for the future as climate change will likely interact with changes in other environmental factors that do not shift across geographic gradients. For example, atmospheric CO_2_ concentrations are projected to continue to increase into the future [37] and might already be responsible for decreasing forage quality in grasslands over the past century [38].

Given the geographic patterns of bison mass and the relatively greater sensitivity to temperature than precipitation, climate change is likely to cause greater nutritional stress for bison and reduce their body size. Whether climate change also affects fecundity has yet to be determined, but the reduced weight gain would likely have a negative effect on economic returns for bison producers. The ecological effects of reduced weight gain, such potential reductions of the amount of grass consumed and nutrient return rates are still uncertain, but will likely affect the ecological roles of bison in native grasslands. That said, there is still much work to do to understand the role climate plays in determining the seasonal timing in dietary quality and weight gain for bison. For example, we do not know the relative importance of climate in determining how much weight is gained during the growing season versus lost during the winter.

The ultimate economic consequences of future climate changes will be dependent on more factors than just grassland condition, but if the magnitude of reductions in weight gain observed for bison transfer to cattle, the economic costs of warming alone could be large. The effects of climate change on domestic cattle are likely to be similar as for bison, but the ecological and economic effects would magnified by over two orders of magnitude. In contrast to the approximately half million bison in North America [39], there are over 100 million cattle in North America. Despite the differences in management and physiology between bison and cattle [39], many of the same principles of the effects of climate on weight gain should transfer. Like bison, cattle growth is frequently limited by protein concentrations [29] and the large majority (>80%) of their caloric intake comes from rangeland, pasture, or other sources of roughage as opposed to cereal crops [40], [41]. If cattle experience similar reductions in weight gain from warming as bison, the costs to US cattle producers of 1°C warming could be in the range of US$1 billion either through direct reductions in weight gain or costs of dietary supplement to compensate for reduced forage quality [4]. In all, assessing the potential effects of climate change needs to directly incorporate slowly-developing processes that affect the dietary quality of forage to grazers, while forage quality and both domestic and native grazer performance need to be directly monitored globally to assess real-time effects of climate change into the future.
